# Approaches and Postoperative Complications of Artery-First Pancreaticoduodenectomy in a Tertiary Care Hospital in Nepal: A Descriptive Cross-sectional Study

**DOI:** 10.31729/jnma.5779

**Published:** 2021-01-31

**Authors:** Roshan Ghimire, Ashik Rajak, Dhiresh Maharjan, Prabin Thapa

**Affiliations:** 1 Department of Surgery, Kathmandu Medical College Teaching Hospital, Kathmandu, Nepal

**Keywords:** *artery first approach*, *early outcome*, *pancreaticoduodenectomy*

## Abstract

**Introduction:**

Superior mesenteric artery first pancreaticoduodenectomy is being increasingly used for pancreatic head and peri-ampullary tumors. The aim of our study was to determine the frequency of various approaches of superior mesenteric artery pancreaticoduodenectomy along with its postoperative complications in a tertiary care center.

**Methods:**

This is a descriptive cross-sectional study of patients undergoing superior mesenteric artery first pancreaticoduodenectomy with different approaches conducted at Kathmandu Medical College Teaching Hospital, Sinamangal, Kathmandu, Nepal, from May 2018 to April 2020. Ethical approval was taken from the Institutional Review Committee (reference no: 310520193). The whole sampling method was adopted. Thirty-four patients undergoing a superior mesenteric artery first pancreaticoduodenectomy at our center with different approaches were included in the study. The data analysis was done in the Statistical Package for the Social Sciences version 20.

**Results:**

For 34 patients chosen for the study, the male: female ratio was 1.6:1, with a mean age of 53.7 years. The medial uncinate approach was done in the majority of the cases, 26 (76.4%), whereas the inferior infracolic (mesenteric) approach was done in 1 (2.9%) case. Regarding postoperative complications, Clavien Dindo grade 3 and grade 4 were present in 11 (32.3%) patients, pancreatic fistula (Grade B and C) was observed in 6 (17.6%) patients, and mortality occurred in 2 (5.8%). The mean hospital stay was 16±9 days.

**Conclusions:**

Superior mesenteric artery first pancreaticoduodenectomy with a different approach can be performed with acceptable morbidity and mortality. Early determination of resectibility is achieved in selected cases.

## INTRODUCTION

The involvement of portal vein or Superior Mesenteric Artery (SMA) was previously considered as an unresectable disease. Gradually with the possibility of vein reconstruction, the involvement of veins was no longer dreaded.

At present, the involvement of >180° of SMA, common hepatic artery, or celiac trunk is considered unresectable. The SMA first approach was proposed as a modification of standard Pancreaticoduodenectomy (PD).^1^ SMA first with different approaches was a new improvement for PD, but there is no evidence whether this approach is advantageous to classical PD or not.^2,3^ SMA first PD allows easier identification, preservation of replaced right hepatic artery and rapidly gives clear information on the posterior extension of the tumor and its resectability.^4,5,6^

There are six well-established approaches for artery first PD.^6^ Our study aimed to explore various approaches of superior mesenteric artery pancreaticoduodenectomy along with its postoperative complications in a tertiary care center.

## METHODS

This is a descriptive cross-sectional study of a prospectively maintained database conducted at Kathmandu Medical College Teaching Hospital, Sinamangal, Kathmandu, Nepal, over two years’ duration from June 2018 to May 2020. Ethical clearance (reference no: 310520193) was obtained from the institute's Institutional Review Board before starting the study. All consecutive patients who underwent SMA first approach pancreaticoduodenectomy over the study period with a minimum of 3 months follow-up were included in the study. Hence, the whole sampling method was used. Only patients planned for artery first pancreaticoduodenectomy were included in the study. Patients who underwent classical and hybrid PD were excluded from the study. The same team of surgeons performed all the surgeries.

All patients were preoperatively assessed with blood samples, tumor markers, CT chest abdomen and pelvis and/or magnetic resonance imaging (MRI) pancreas. Pre-operative variables included age, gender, body mass index, and indication for surgery. Intra-operative variables included operative time, blood loss, and transfusion requirements. Postoperative data and complications were recorded and described according to the Clavien-Dindo classification. Pancreas-specific complications were assessed and graded according to the International Study Group's recommendations on Pancreatic Surgery.^7^ Continuous data were expressed as mean ± standard deviation (SD), and categorical data were expressed as a percentage. Data were analyzed using the Statistical Package for the Social Sciences (SPSS) version 20. It was ensured that participants gave informed consent before enrolling in the study.

## RESULTS

Over two years, from June 2018 to May 2020, 34 patients underwent SMA first approach PD. The patients’ mean age in this study was 53.7 years, with a male: female ratio of 1.6:1 (Table 1). Table 1 also shows various perioperative variables in SMA first approach pancreaticoduodenectomy cases.

**Table 1:** Patient demographics and perioperative variables in SMA first approach pancreaticoduodenectomy cases.

Patient Demographics	Value
Mean age (years)	53.7
Sex (Male: female)	1.6:1
Perioperative variables	Mean SD (range)
Operating time (minutes)	279±59 ( 221-338)
Blood loss(ml)	750±450 (300-1200)
PRBC transfusion requirement(units)	3±3 (0-6)
ICU stays (days)	8±4 (4-12)
Hospital stay (days)	16± (7-26)

Patient indications for surgeryin SMA first approach pancreaticoduodenectomy cases are listed (Table 2).

**Table 2:** Patient indications for surgery in SMA first approach pancreaticoduodenectomy cases.

Indications for surgery	Frequency n (%)
Uncinate mass	10 (29.4)
Ampullary growth	12 (35.9)
Distal cholangiocarcinoma	7 (20.58)
Chronic pancreatitis	1 (2.9)
Groove pancreatitis	2 (5.8)
Pancreatic cystic neoplasm	2 (5.8)
Total	34 (100)

The medial uncinate approach was done in the majority of the cases, 26 (76.4%), whereas the inferior infracolic (mesenteric) approach was done in 1 (2.9%) case (Table 3).

**Table 3:** Different approaches of SMA pancreaticoduodenectomy.

Approaches	Value n (%)
Medial uncinate approach	26 (76.4)
Left posterior approach	2 (5.8)
Posterior approach	3 (8.8)
Inferior supracolic (anterior) approach	2 (5.8)
Inferior infracolic (mesenteric) approach	1 (2.9)
Superior approach	0 (0)
Total	34 (100)

Regarding postoperative complications, Clavien Dindo grade 3 and grade 4 were present in 11 (32.3%) patients, pancreatic fistula (Grade B and C) was observed in 6 (17.6%) patients, and mortality occurred in 2 (5.8%) (Table 4).

**Table 4:** Postoperative complications of SMA first pancreaticoduodenectomy.

Postoperative complications	Frequency n (%)
Post-operative pancreatic fistula (POPF)	6 (17.6)
Grade B	5 (14.7)
Grade C	1 (2.9)
Delayed gastric emptying (DGE)	8 (23.5)
Grade A	6 (17.6)
Grade B	2 (5.8)
Grade C	0 (0)
Re-exploration	3 (8.8)
Pseudo-aneurysm	2 (5.8)
Wound complications	10 (29.4)
Superficial	9 (26.4)
Deep	1 (2.9)
Pulmonary complications	3 (8.8)
Cardiac complications	1 (2.9)
Others	5 (14.7)
Mortality	2 (5.8)

The overall postoperative morbidity was seen in 18 (52.9%) cases as per the Clavien-Dindo classification scheme with major complications (Clavien-Dindo grades 3 and 4) occurring in 11 (32.35%) cases. Five patients had more than one complication.

The clinically significant fistula rate (Grade B and C) was seen in 6 (17.6%) cases. The incidence of postpancreatectomy hemorrhage was seen in 2 (5.8%) cases. Three patients required re-exploration, two for immediate intra-abdominal hemorrhage and one for grade C pancreatic fistula.

On re-exploration for post-pancreatectomy hemorrhage, bleeding was identified from the retroperitoneal blood vessel in one case, suture-ligated, and in the next case, a large clot was present intra-abdominally. However, no active bleeding vessel was identified. One patient had re-operation for pancreatic fistula (grade C fistula) and underwent replacement of drains after drainage of peri-pancreatic collection. Two patients developed pseudo-aneurysm of the gastroduodenal artery, which was managed with coiling.

The overall 30-day mortality rate was seen in 2 (5.8%) cases in this study. Out of two cases, one patient required re-intubation for respiratory insufficiency with right-sided lower zone pneumonia on 16^th^ postoperative day, which later expired on day 19, whereas the second patient died due to sepsis because of grade C pancreatic fistula on the 29^th^ postoperative day, which was re-explored on the 9^th^ postoperative day.

## DISCUSSION

This study was done to determine the frequency of various approaches and postoperative complications following Pancreaticoduodenectomy procedure, SMA first approach in a tertiary care center in Nepal. Overall postoperative morbidity was seen in 52.9% of cases in our study, with major complications (Clavien-Dindo grade 3 and 4) occurring in 32.35% of cases.

Since its inception in the early twentieth century, pancreaticoduodenectomy has seen many modifications. With the change in surgery techniques, there has been a change in understanding, prognosis, and overall disease concept. Artery first approach is thought to have the advantage of identifying any tumor-infiltrating to SMA before proceeding to other steps. This approach has shown the least operating time too.^3^ In classical PD, the dissection of SMA usually is done at a later stage, and access for reconstruction is also limited. However, in the artery first technique, SMA involvement is identified for early determination of unresectability. Though unresectability can be determined early on the basis of imaging, it might differ during operative assessment in a neoadjuvant setting due to fibrosis and stranding.^8,9^ In literature, different methods to artery first approach has been defined. There are six well-established approaches, namely, from the retroperitoneum (posterior approach), the uncinate process (medial uncinate approach), the infracolic region medial to the duodenojejunal flexure (inferior infracolic or mesenteric approach), the infracolic retroperitoneum lateral to the duodenojejunal flexure (left posterior approach), the supracolic region (inferior supracolic approach) and through the lesser sac (superior approach).^6^

The main objective of PD is to obtain an R0 margin. If the initial step in surgery shows a possibility of achieving this goal, only then it is rational to proceed. SMA first is a safe technique. The SMA-PD is associated with better perioperative outcomes, such as blood loss, transfusion requirements, pancreatic fistula, and delayed gastric emptying.^1,8,9^ Rate of complications and mortality in this study is somehow comparable with other series and with standard classical PD series.^3,4^ Study done by Kumar SV and Prasad AS morbidity was seen in 55%, comparable with our study.^4^ Mortality of our study (5.8%) is comparable with that of a study done by Pal S et al. (10%) where he has observed 5.6% mortality among the SMA first group.^3^ The patient's prognosis is determined ultimately with tumor biology and achievement of the negative resection margin.^10^ Irrespective of whichever approach is performed, the goal of surgery is to decrease perioperative morbidity with the maintenance of oncological principal.^11^

The medial uncinate first approach is useful for bulky tumors arising in the superior aspect of the pancreatic head facilitating the alignment of the uncinate process with the jejunal mesentery, which ultimately allows the complete dissection of the SMV and SMA.^12,13^ Majority of the cases, 76.4% have been done with this approach among which two patients had cystic neoplasm of the pancreas.

Posterior approach PD enables complete dissection of the superior mesenteric artery's right side, portal vein with complete excision of the retro-portal pancreatic process.^14,15^ In this study, 8.8% of cases were approached with this maneuver. The first case had replaced the right hepatic artery, whereas the other two had portal vein infiltration requiring portal vein reconstruction with end-to-end anastomosis. These two cases did well in the postoperative period with prophylactic management by low molecular weight heparin.

The left posterior approach allows adequate assessment of the SMA without the mobilization of the duodenum or colon and is particularly useful for tumors arising from the uncinate and posterior aspect of the head of the pancreas.^16^ This approach was used in 5.8% of cases, and both cases were diagnosed as groove pancreatitis. One case developed postoperative diarrhea, which was managed with anti-diarrheal agents.

The inferior supracolic (anterior) approach is useful in lower pancreatic head tumors, and it allows the assessment of arterial involvement at an early stage of the operation.^17^ 5.8% cases were done with this approach. However, the early division of the stomach and pancreatic neck is debatable in this maneuver.

Inferior infracolic (mesenteric) maneuver allows early determination of SMA involvement without handling the pancreatic head and tumor. This approach facilitates the early ligation of the inferior pancreatico-duodenal artery and allows dissection of the region posterior to the SMA under direct vision.^18^ This approach was only used in 2.9% of cases where SMV and middle colic vein were involved. Vascular reconstruction with end-to-end anastomosis of SMV was done. The patient did well in the postoperative period with prophylactic low molecular weight heparin.

SMA's first PD approach helps in the early identification of various hepatic arterial aberrations and preserve them. Vascular control and, when required, vascular resection and reconstruction look feasible with this approach.^5,6^ In this study, reconstruction of SMV was done with inferior infracolic approach, and two portal vein reconstructions were done with posterior approach SMA first PD.

This study included a heterogeneous group of patients, including benign pathology, and was conducted in a single center with limited sample size. Hence, the findings of our study can't be generalized. Further larger multicentric studies that look into the association between variables and those which address confounders should be done to gain more insight.

## CONCLUSIONS

Superior mesenteric artery first pancreaticoduodenectomy with different approaches is safe and effective in pancreatic resections. SMA first PD improves safety as it facilitates a better understanding of the superior mesenteric vascular pedicle anatomy.
